# *Burkholderia cepacia* Complex Lumbar Spondylodiscitis: A Rare Nosocomial Infection

**DOI:** 10.1155/2022/4378442

**Published:** 2022-02-16

**Authors:** Rachel Subramanian, Lynn Fitzgibbons

**Affiliations:** ^1^Department of Medical Education, Santa Barbara Cottage Hospital, Santa Barbara, CA, USA; ^2^Department of Infectious Diseases, Santa Barbara Cottage Hospital, Santa Barbara, CA, USA

## Abstract

Pyogenic spondylodiscitis is rarely caused by *Burkholderia cepacia* complex. *B. cepacia* is widespread in the environment and recognized as an opportunistic pathogen for patients with cystic fibrosis and immune disorders. A female in her mid-30s with underlying hyperthyroidism, but otherwise immunocompetent, was admitted to the hospital with persistent lower back pain after elective bariatric surgery in Mexico. Lumbar MRI showed L2/L3 osteomyelitis and discitis. Culture of disk aspiration grew *Burkholderia cepacia* complex sensitive to cefepime, ceftazidime, ciprofloxacin, gentamicin, imipenem, levofloxacin, and trimethoprim-sulfamethoxazole. The infection failed to respond to cefepime; however, she was successfully treated with levofloxacin monotherapy.

## 1. Introduction

Pyogenic spondylodiscitis is an infection of the intervertebral discs and/or adjacent vertebrae. It occurs by hematogenous seeding of bacteria, direct spread from a nearby infection, or from direct inoculation during spinal surgery [1].

The most common pathogen identified is *Staphylococcus aureus*, followed by Gram-negative rods, coagulase-negative staphylococci, streptococci, enterococci, and anaerobes. In many cases, an organism is not identified. The diagnosis is based on clinical, radiological, and microbiological findings [2].

The diagnosis of pyogenic spinal infection is often delayed because of its insidious presentation and nonspecific signs and symptoms. While the most common initial complaint is back or neck pain in 90% of cases, fever and neurological involvement are rarely present [1]. Diagnostic delay is common, especially in a patient without risk factors, but prompt diagnosis and treatment are critical because pyogenic spondylodiscitis can lead to spinal instability and neurologic complications [2].

*Burkholderia cepacia* complex *(B. cepacia)* is a Gram-negative bacillus known to infect immunocompromised patients. This microorganism is notoriously hard to target because of its multidrug-resistant nature [3]. Here, we report a case of *Burkholderia cepacia* complex lumbar pyogenic spondylodiscitis after bariatric surgery in an otherwise healthy patient. She failed treatment with cefepime but achieved clinical remission with levofloxacin monotherapy.

## 2. Case Presentation

A 34-year-old female with a past medical history of obesity (body mass index: 33) and Graves' disease presented to the emergency room with progressive lower back pain and difficulty walking. The pain initially started four days after an elective laparoscopic sleeve gastrectomy performed in Tijuana, Mexico. The patient was seen in the emergency department one day after pain onset for significant lower back pain radiating to her right leg. She was diagnosed with musculoskeletal pain and sciatica and then discharged on oral morphine. Ten days later, she visited her primary care provider for the same lower back pain and was started on gabapentin. A lumbosacral X-ray and lumbar magnetic resonance imaging (MRI) were ordered, but never completed.

**Figure 1:**
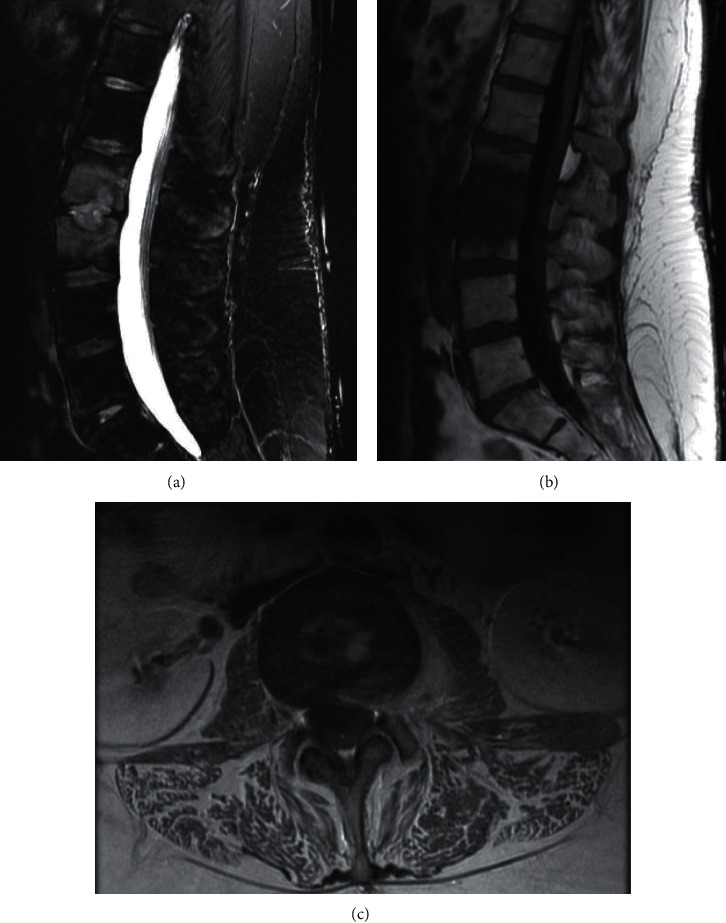
Sagittal T2-weighted (a), T1-weighted (b), and axial T1-weighted postcontrast (c) images demonstrating signal abnormality within the L2 and L3 vertebral bodies adjacent to the intervertebral disk space, consistent with osteomyelitis and discitis, with an extension to the left psoas muscle and epidural space.

She returned to the emergency room within one week of her primary care visit for worsening back pain, paresthesia affecting the anterior thighs, and impaired gait. Vital signs were blood pressure 149/97 mmHg, heart rate 132 beats per minute, temperature 36°C, respiratory rate 18 breaths per minute, and oxygen saturation 100% on room air. Physical examination was notable for point tenderness over the lumbar spine region. Neurological exam was otherwise normal. Laboratory results demonstrated white blood cell count 6,300/*µ*L (normal: 4,000–11,000/*µ*L), C-reactive protein (CRP) 36 mg/L (normal: <10 mg/L), erythrocyte sedimentation rate (ESR) 90 mm/Hr (normal: <20 mm/Hr), and procalcitonin 1.6 ng/mL (normal: <0.5 ng/mL), compatible with infection. She denied postoperative complications, intravenous drug use, or immunosuppressive medications. She was prescribed methimazole for hyperthyroidism but had not taken it since undergoing surgery. Her HIV test was negative. Spinal MRI with contrast showed L2/L3 early discitis and osteomyelitis without a fluid collection or abscess (Figure 1).

A fluoroscopically guided aspiration of the L2/L3 disk was performed by injecting a 1 cc sterile saline wash which was aspirated and sent for culture. *Burkholderia cepacia* complex grew on chocolate agar and was identified using matrix-assisted laser desorption/isolation time-of-flight mass spectroscopy (MALDI-TOF MS). The sensitivities were determined with the VITEK system, which showed resistance to piperacillin-tazobactam and susceptibility to cefepime, ceftazidime, ciprofloxacin, gentamicin, imipenem, levofloxacin, and trimethoprim-sulfamethoxazole. Cytology showed mild to moderate neutrophils. Blood and urine cultures remained negative.

She was treated initially with vancomycin and ceftriaxone awaiting culture results and then switched to six weeks of cefepime 2 g every 12 hours via a peripherally inserted central catheter. Four weeks later, the patient returned to the hospital for unrelenting lower back pain and increasing inflammatory markers. Spinal survey MRI showed worsening L2/L3 osteomyelitis. A repeat intervertebral aspiration grew *Burkholderia cepacia* complex susceptible to levofloxacin. Levofloxacin 750 mg IV was given for six weeks; ESR normalized, and CRP decreased to 11 mg/L. Lumbar spine MRI was repeated after completion of antibiotics. Imaging showed improvement of discitis and osteomyelitis at L2/L3 and no drainable fluid collection. The patient reported intermittent lower back pain which could be explained by the interval development of subacute mild compression fractures of the superior endplates of L1, L4, and L5.

## 3. Discussion

Originally described in 1950 by an American microbiologist William Burkholder as the causative agent of onion rot, *Burkholderia cepacia* complex is a motile, catalase-producing, and nonlactose-fermenting aerobic Gram-negative bacillus. This bacterium is found ubiquitously in the environment, dwelling in water, soil, and various plants, where it thrives in moisture [3]. *B. cepacia* is known for infecting the respiratory tracts of patients with cystic fibrosis (CF) and chronic granulomatous disease, resulting in a wide range of clinical manifestations from chronic asymptomatic colonization to bacteremia and necrotizing pneumonia [4, 5].

Our patient developed osteomyelitis within one month postoperatively after bariatric surgery in Mexico. While the source of the patient's infection remains unknown, the development of symptoms soon after surgery raises suspicion for an infection of nosocomial origin. In recent years, immunocompetent patients have become infected with *Burkholderia cepacia* complex in healthcare settings [4]. There are reports of intensive care unit outbreaks of this pathogen after exposure to contaminated antiseptics, disinfectants, dextrose solutions, nebulizer solution, sublingual probes, nasal spray, mouthwash, chlorhexidine, tap water, enteral feeding, and ultrasound gel [3]. As a result, infections such as meningitis, pneumonia, endocarditis, septic arthritis, peritonitis, osteomyelitis, and bacteremia have been reported in the literature [6].

*B. cepacia* bacteria cause such significant morbidity and mortality in patients because of their various resistance mechanisms to overcome the action of antimicrobials. These include beta-lactam resistance, efflux pump-mediated multidrug resistance, outer membrane permeability barrier, and alterations in drug targets. Such mechanisms lead to innate resistance to antipseudomonal penicillins, polymyxin B, and aminoglycosides [7]. The recommended first-line therapy is trimethoprim and sulfamethoxazole, but meropenem, ceftazidime, fluoroquinolones, chloramphenicol, piperacillin, and minocycline, alone or in combination, are considered appropriate antimicrobial treatments [8].

Cefepime given intravenously twice daily was chosen because it distributes widely in most body tissues and fluids. Twice daily dosing is also more feasible than three times per day as an outpatient. The pharmacokinetic profile of two grams of cefepime demonstrates excellent cortical and cancellous bone penetration [9]. Intravenous as opposed to oral antibiotics were selected because of the concern for unpredictable bioavailability of beta-lactams and fluoroquinolones after bariatric surgery [10]. For the minority of patients who do not respond to antibiotics alone, the next step in treatment may be spinal stabilization or surgical debridement. Indications for surgery include the development of neurological deficits or spinal cord compression, spinal instability, presence of epidural or paravertebral abscesses, or evidence of progression despite appropriate antimicrobial therapy [11]. In our case, *B. cepacia* did not respond to cephalosporins (despite being sensitive); however, the infection was eliminated with levofloxacin.

Pyogenic spondylodiscitis caused by *Burkholderia cepacia* complex is a very uncommon entity; few cases are reported in the literature. Our patient is unique because she had no predisposing risk factors for pyogenic spondylodiscitis but developed osteomyelitis and discitis at L2/L3. Among patients with Graves' disease, there is no established increased susceptibility to *B. cepacia* infections. The risks of osteomyelitis include chronic kidney disease, malnutrition, substance abuse, HIV, diabetes mellitus, long-term steroid use, liver cirrhosis, and malignancy [1]. This case is also remarkable because *B. cepacia* rarely causes osteomyelitis and discitis. Most cases are related to spinal surgery, trauma, intravenous drug use, or postoperative complications [4, 12–16]. One case-control study of non-CF patients found recent abdominal surgery to be an independent risk factor for *Burkholderia cepacia* bacteremia [17].

In summary, *Burkholderia cepacia* complex is an under-recognized, but important cause of pyogenic spondylodiscitis in immunocompetent patients. Due to the debilitating nature of this disease and multidrug resistance of this bacterium, a swift diagnosis and determination of susceptibility are paramount for treatment. If left untreated or treated with an inappropriate antibiotic, spinal infections may spread to adjacent structures in the spinal canal and cause abscess formation in the epidural space and in soft tissues and muscles. This can lead to destruction of the intervertebral disk and vertebral bodies, ultimately causing spinal cord compression, paralysis, and death [2].

## 4. Conclusion

Pyogenic spondylodiscitis is rarely caused by *Burkholderia cepacia* complex. Our case reports a patient with *B. cepacia* lumbar osteomyelitis and discitis after bariatric surgery. This case highlights how critical it is to obtain a Gram stain and culture with sensitivities when selecting antibiotics, especially for microorganisms with inherent resistance mechanisms. Although uncommon in immunocompetent patients, *B. cepacia* is an important emerging nosocomial pathogen which should be considered in the differential for osteomyelitis after surgery.
